# Physiologic Transdermal Estradiol Replacement Mimics Effects of Endogenous Estrogen on Bone Outcomes in Hypoestrogenic Women with Anorexia Nervosa

**DOI:** 10.3390/nu14132557

**Published:** 2022-06-21

**Authors:** Vibha Singhal, Supritha Nimmala, Meghan Slattery, Kamryn T. Eddy, Karen K. Miller, Anne Klibanski, Madhusmita Misra

**Affiliations:** 1 Neuroendocrine Unit, Massachusetts General Hospital and Harvard Medical School, 55 Fruit Street, Boston, MA 02114, USA; snimmala@mgh.harvard.edu (S.N.); mslattery@mgh.harvard.edu (M.S.); keddy@mgh.harvard.edu (K.T.E.); kkmiller@mgh.harvard.edu (K.K.M.); aklibanski@partners.org (A.K.); mmisra@mgh.harvard.edu (M.M.); 2 Division of Pediatric Endocrinology, Massachusetts General Hospital and Harvard Medical School, 55 Fruit Street, Boston, MA 02114, USA; 3 MGH Weight Center, Massachusetts General Hospital and Harvard Medical School, 55 Fruit Street, Boston, MA 02114, USA; 4 Eating Disorders Clinical and Research Program, Massachusetts General Hospital, Boston, MA 02114, USA

**Keywords:** estradiol, anorexia nervosa, adolescents, bone density, bone structure

## Abstract

Background: While physiologic estrogen replacement results in increases in areal bone mineral density (aBMD) in hypoestrogenic adolescent girls and young adult women with AN, data are lacking regarding its impact on measures of volumetric BMD (vBMD), bone geometry, and structure. Methods: 23 young women with anorexia nervosa (AN) and 27 normal-weight healthy controls (HC) between 14–25 years old were followed for 12 months. AN participants received transdermal 17β-estradiol (continuously) with 10 days of cyclic oral progesterone (100 mg daily) every month for the study duration (AN-E+). DXA was used to measure aBMD and body composition, high resolution peripheral quantitative CT (HRpQCT) to assess vBMD, bone geometry and structure at the distal radius and tibia, and microfinite element analysis to estimate strength. Results: Groups did not differ for age. Median baseline BMI z-scores were −1.13 (−1.58, −0.38) in AN-E+ vs. 0.08 (−0.40, 0.84) in HC (*p* < 0.0001). For most HRpQCT parameters and strength estimates, young women with AN receiving physiologic estrogen replacement demonstrated similar changes over 12 months as did normoestrogenic HC. Additionally, radial cortical tissue mineral density, cortical vBMD, and failure load increased (*p* = 0.01; *p* = 0.02; *p* = 0.004 respectively) over 12 months in AN-E+ compared to HC. Conclusions: With physiologic estrogen replacement, bone accrual improved in AN to approximate changes observed in normoestrogenic controls followed without any intervention, with additional benefits observed for cortical tissue mineral density, cortical vBMD, and failure load at the radius in AN vs. controls. Thus, this strategy for estrogen replacement effectively mimics the effects of endogenous estrogen on bone structure and estimated strength.

## 1. Introduction

Anorexia nervosa (AN), an eating disorder characterized by weight loss from persistent food restriction and body image distortion, is associated with low bone mineral density (BMD) and reduced bone strength [1,2]. Due to the rapid bone accrual that occurs during adolescence, this period is critical for optimizing peak bone mass [3]. Reduced bone accrual rates in adolescents with AN [4,5] raise concerns for long-standing deficits in bone health and increased fracture risk [6].

Low energy availability states, as seen in AN, often result in hypogonadism due to suppression of the hypothalamic-pituitary-gonadal axis causing low estrogen levels. Since the broadening of the diagnostic criteria for AN in the Diagnostic and Statistical Manual of Mental Disorders Fifth Edition (DSM-5), amenorrhea is no longer required for the diagnosis of this condition. However, menstrual irregularities are frequently observed in these young women. Estrogen is a key regulator of bone metabolism, and hypoestrogenism in AN is an important determinant of impaired bone accrual, low areal (aBMD), and volumetric BMD (vBMD), and impaired bone geometry and structure [7,8,9].

In adolescents with AN, weight gain and menstrual recovery halt further deterioration of aBMD at the lumbar spine and whole body, as assessed by dual energy X-ray absorptiometry (DXA) [4]. Furthermore, physiologic estrogen replacement (unlike the use of a combined oral contraceptive pill) in adolescents with AN increases bone accrual at the spine and hip (assessed using DXA) to approximate accrual rates observed in normal-weight healthy controls [10]. Data are currently lacking regarding the impact of physiologic estrogen replacement in young women with AN on specific bone compartments and measures of vBMD, bone geometry and structure (key contributors to bone strength) as measured by high resolution peripheral quantitative computed tomography (HR-pQCT).

Our objective was to compare changes in vBMD, bone geometry, and structure in adolescents and young adult women with AN who received physiologic estrogen replacement as transdermal 17β-estradiol (17β-E2) in replacement doses (with cyclic oral progesterone) over 12 months (AN-E+) to changes observed in normoestrogenic healthy controls (HC) over 12 months. We hypothesized that with physiologic estrogen replacement in AN, changes in these bone parameters would approximate changes observed in HC, who have normal endogenous estrogen and are followed without any intervention.

## 2. Participants and Methods

### 2.1. Participant Selection

23 adolescent and young adult oligo-amenorrheic women with AN and 27 normal-weight, normally menstruating, healthy controls (HC) aged 14–25 years, were enrolled. A bone age assessment of 14 years and above was a prerequisite for participants to be included in the study. Prior to enrollment, participants with AN/atypical AN met the DSM-IV or DSM-5 criteria for the diagnosis of this condition (depending on whether recruitment occurred before or after publication of DSM-5), as confirmed by the study psychologist. Low weight criteria for those with AN required them to be below 90% for two of the following three measures based on Centers for Disease Control (CDC) growth charts for girls [11]: (i) percentage median body mass index (% mBMI) for age, (ii) percentage median body weight (% mBW) for age, or (iii) percentage median body weight for height (% mBW-Ht). Participants were encouraged to initiate or abide by treatment for AN and its concomitant comorbidities as instructed by their eating disorder providers or treatment team, as the study did not assume clinical care for the AN participants. Exclusion criteria included standard contraindications to estrogen therapy, a history of medical conditions that are reported to affect bone metabolism or menstrual status such as Cushing’s syndrome, diabetes mellitus, pituitary disease, renal failure, untreated thyroid disease (assessed using a thyroid stimulating hormone (TSH) level), or primary ovarian insufficiency (assessed using a follicle stimulating hormone (FSH) level), a history of bone fracture in the six months prior to enrollment in the study (as this may impact reporting of bone outcomes), and current or previous history of consumption of medications reported to impact bone metabolism (such as bisphosphonates or long-term steroids). We did not exclude participants with a current or previous history of usage of oral medications containing estrogen or compounds containing progestin given the limited impact of these medications on BMD in AN [12]. However, if usage of such medications was reported by participants at the time of the screening visit, they were asked to discontinue these medications for a minimum of sixty days prior to the baseline visit. Further, to minimize risk from study participation, we excluded participants with a diagnosis of pregnancy, suicidality, substance abuse, psychosis, hematocrit below 30% (indicative of anemia), and potassium below 3.0 mmol/L (suggestive of active purging). Participants with AN were enrolled in a study (R01 DK062249) evaluating the impact of adding recombinant human insulin-like growth factor-1 (rhIGF-1) to physiologic estrogen replacement with respect to bone outcomes in adolescent and young adult women with AN [13]. Only participants who received physiological estrogen (and placebo rather than rhIGF-1) were included in the current analysis. Healthy control participants were drawn from two studies (R01 DK062249, R0I HD060827), and were required to meet inclusion/exclusion criteria for the current analysis.

### 2.2. Study Protocol

The study was approved by the Institutional Review Board of Partners HealthCare and is Health Insurance Portability and Accountability Act compliant. Informed consent was obtained from participants ≥18 years. If participants were <18 years old, informed consent was obtained from the parents and informed assent was obtained from participants. Study visits were conducted at the Translational and Clinical Research Center of our institution. The screening visit included a detailed medical history and physical examination, urine pregnancy test, and fasting blood draw to rule out causes of amenorrhea other than hypothalamic amenorrhea. Weight of participants (in a hospital issued gown) was measured on an electronic weighing scale to the closest 0.1 kg. A wall-mounted stadiometer was used to obtain their height to the closest 0.1 cm, and the mean of three measurements was recorded. Bone age was determined by an X-ray of the left hand and wrist. To prevent premature epiphyseal fusion in girls who were actively growing, only girls with a bone age of 14 years and above were included. Following the screening visit, the baseline visit was scheduled to occur within 60 days. At the baseline visit, participants were asked in detail about the average hours of physical exercise (weight-bearing and non-weight-bearing) per week. All participants underwent a fasting blood draw (see Laboratory Measures), a DXA scan, and an HRpQCT scan at baseline and then at 12-months. All AN participants received 17β-E2 at a dose of 0.1 mg/day delivered transdermally twice weekly continuously (AN-E+), with 100 mg progesterone given for 10 days of each month (to avoid unopposed estrogen administration causing endometrial hyperplasia).

### 2.3. Areal Bone Mineral Density (aBMD) and Body Composition Assessments

We used DXA (Dual-energy X-ray Absorptiometry; Hologic 4500 A, Apex software version 13.3; Hologic Inc., Waltham, MA, USA) to measure aBMD of the femoral neck, total hip, lumbar spine, and whole body, and total fat and lean mass (measures of body composition) for each subject. At our institution, the coefficients of variation for aBMD, fat mass, and lean mass measurements are 0.8% to 1.1%, 2.1%, and 1.0%, respectively. For participants 18 years and below the standard pediatric database was utilized for calculation of BMD Z-scores, and for participants 19 years and above the standard adult database was used [14]. By employing the same database throughout the course of the 12-month duration of the study, consistency of assessments was maintained

### 2.4. Volumetric Bone Mineral Denisty (vBMD), Bone Geometry and Structure Assessment

We used HRpQCT imaging (XtremeCT; Scanco Medical AG, Bassersdorf, Switzerland) to assess vBMD, bone geometry, and structure of the non-dominant distal radius and tibia. Tissue mineral density (TMD), thickness, and porosity of cortical bone were obtained by extended cortical analysis (ECA), and micro finite element analysis (µFEA) was used to obtain strength estimates (stiffness and failure load) [15,16]. As long as there was no previous history of fracture on the non-dominant side, the scan was obtained on that side; the non-fractured dominant side was assessed in case of a previous history of fracture (acute) in the non-dominant side. The slices were measured at 9.5 mm from the radial endplate and 22.5 mm from tibial end plate by employing 2D scout views as a part of the scan protocol. All participants had DXA and HRpQCT data at both timepoints except for two AN participants and one HC who did not have HRpQCT scans for the distal radius due to motion artifact, which rendered them unusable for determining bone endpoints.

### 2.5. Laboratory Measures

Our hospital laboratory assessed FSH, TSH, hematocrit, potassium, and glucose levels, which comprised the screening labs. For other labs, serum or plasma was stored at −80 degrees Celsius until the end of the study, when samples were analyzed using the following assays. Radioimmunoassay was used to assess N-terminal propeptide of type 1 procollagen (P1NP), a bone formation marker, (Orion Diagnostics, Espoo, Finland; sensitivity 0.7 μg/L; intraassay coefficient of variation (CV) 3.5–5.3%). N-telopeptide (NTX), a bone resorption marker, was assessed with an enzyme immunoassay (Alere Osteomark, Scarborough, ME, USA; sensitivity 5 nM BCE; intraassay CV 4.6%). Calcium and phosphorus levels were estimated by a colorimetric assay (LabCorp Esoteric Testing, Burlington, NC, USA; sensitivity 0.8 mg/dL; intraassay CV 0.9–3.0% for calcium, and sensitivity 6.0 pg/mL; intraassay CV 0.9–3.0% for phosphorus), and 25 hydroxy vitamin D (25OHD) levels were measured by an immunochemiluminometric assay (LabCorp Esoteric Testing, Burlington, NC, USA; sensitivity 4.0 ng/mL; intraassay CV 4.8–7.7%). A chemiluminescent immunoassay was used to assess parathyroid hormone (PTH) levels (Beckman Coulter, Fullerton, CA, USA; sensitivity 1 pg/mL; intraassay CV 1.6–2.6%). Liquid chromatography/mass spectrometry (LC/MS) estimated insulin like growth factor-I (IGF-I) (Quest Diagnostics, Nichols Institute, San Juan Capistrano, CA, USA; sensitivity 15.6 ng/mL; intraassay CV 3.5–15%). Estradiol levels were measured by chemiluminescence (Beckman Coulter, Fullerton, CA, USA; sensitivity 20 pg/mL; intraassay CV 2.0–4.2%).

### 2.6. Statistical Analysis

Mean ± SEM for parametric data or median with interquartile range for non-parametric data were the formats used to report data after assessing for normality using the Shapiro Wilk test. Student *t*-test (for parametric data) or Wilcoxon rank sum test (for non-parametric data) for between group analysis was used based on data distribution. The paired *t*-test (parametric data) or Wilcoxon signed-rank test (non-parametric data) was used for within group changes. For distributions that were right skewed, we performed a log transformation (cortical vBMD and failure load at the radius). All bone endpoints are reported after controlling for age and race. Multivariate analysis was used to control for age, race, and change in weight over the study duration.

## 3. Results

### 3.1. Baseline Characteristics and Changes in Clinical Characteristics, and Body Composition over 12 Months

AN-E+ and HC groups were similar for age and bone age. AN-E+ had a mean age of 16.23 ± 0.83 years at the time of AN diagnosis. The mean age of menarche was higher in AN-E+ compared to HC. AN-E+ had a mean duration of 7.77 ± 2.72 months of amenorrhea. As expected, AN-E+ had lower weight, BMI, BMI z-scores, percent mBMI, and total and percent fat mass than HC, while percent lean mass and weight-bearing activity were higher in AN. Over the course of 12 months, AN-E+ demonstrated greater increases in percent mBMI, total lean mass and percent fat mass compared with HC. Within the AN-E+ group, increases were observed in weight, BMI, and total lean mass (Table 1).

### 3.2. Baseline Characteristics and 12-Month Change in Biochemical Parameters

Table 2 reports baseline bone parameters and changes over 12-months.

Bone turnover markers: P1NP levels were similar in the AN-E+ and HC groups at baseline. However, HC demonstrated a within group reduction in P1NP at 12 months. NTX was similar between groups at baseline, but a within group reduction was noted in AN-E+ at 12 months.

Calcium, 25OHD and PTH: At baseline, AN-E+ had higher levels of calcium and 25OHD, and lower levels of PTH than HC. Over 12 months, we observed a decrease in calcium and 25OHD levels in AN-E+ and an increase in HC. However, levels of both remained within the normal clinical range at 12 months.

IGF-I and Estradiol: For between group comparisons, baseline IGF-I levels (and corresponding Z-scores), and changes in these levels over 12 months did not differ in AN-E+ vs. HC groups. We did observe a within group decrease in IGF-I levels and IGF-I Z-scores over 12 months in the HC group. At baseline, AN-E+ had a median estradiol level of 45.2 (10.5, 56.6) pg/mL. As expected, with transdermal 17β-E2 supplementation in AN-E+, levels of estradiol increased at 12 months to 134.0 (69.5, 169.1) pg/mL (*p* < 0.0001).

### 3.3. Baseline Characteristics and 12-Month Changes in DXA Measures of Areal BMD

**Figure 1:**
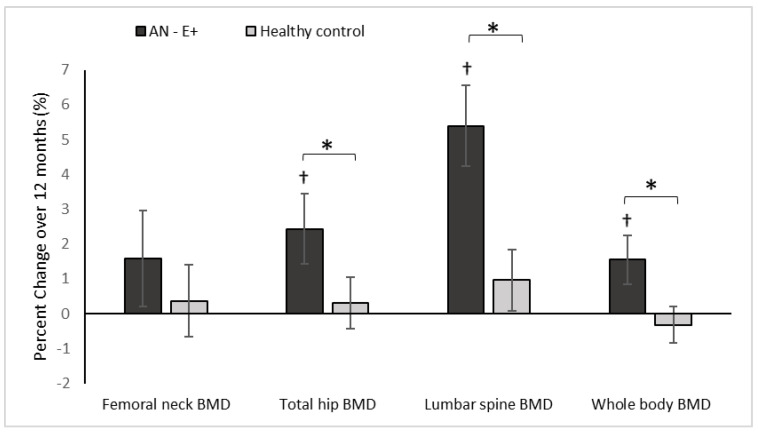
Percent change in DXA measures of areal BMD (bone mineral density) in the AN-E+ and healthy control groups over 12-months, * *p* < 0.05 for between group comparison after adjusting for age and race, † *p* < 0.05 for within group change.

Table 3 reports absolute changes in DXA endpoints over 12 months, while Figure 1 demonstrate percent changes in these endpoints over the study duration.

At baseline, AN-E+ group had lower aBMD and aBMD Z-scores at the femoral neck, total hip, and lumbar spine, and whole body compared to the HC group, which remained significant after controlling for age and race. With physiologic estrogen replacement administered as transdermal 17β-E2 over 12 months, AN-E+ demonstrated increases in aBMD at the lumbar spine and whole body compared to HC (controlled for baseline age and race) and a trend for an increase was noted at the total hip. After adjusting for age, race and weight change, increments in lumbar spine aBMD and whole body aBMD were greater in AN-E+ compared to HC. Within the AN group, increases in aBMD at the total hip, lumbar spine, and whole body, and increases in aBMD Z-scores at the femoral neck, total hip, and lumbar spine were noted.

### 3.4. Baseline Characteristics and 12-Month Change in HRpQCT Bone Parameters

**Figure 2:**
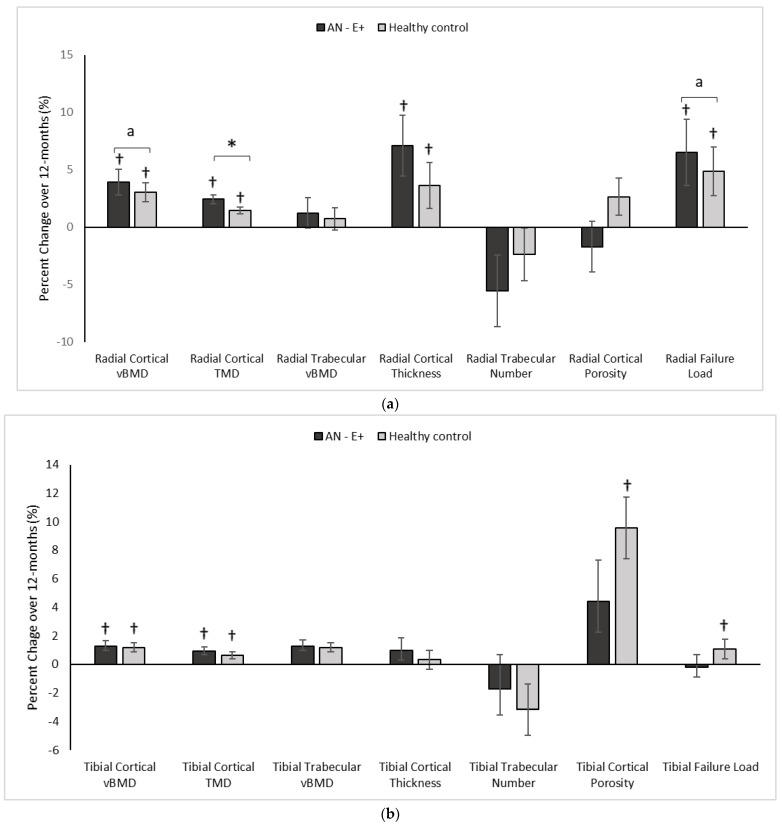
(**a**) Percent change in Radial HRpQCT parameters in the AN-E+ and healthy control groups over 12-months, * *p* < 0.05 for between group comparison after adjusting for age and race, a *p* < 0.05 for between group comparisons on log conversion for right skew of data distribution, † *p* < 0.05 for within group change. (**b**) Percent change in Tibial HRpQCT parameters in the AN-E+ and healthy control groups over 12-months, * *p* < 0.05 for between group comparison after adjusting for age and race, † *p* < 0.05 for within group change. TMD: Tissue mineral density. a indicates *p* < 0.05 for between group comparisons on log conversion for right skew of data distribution.

Table 4 reports absolute changes in HRpQCT and µFEA endpoints over 12 months, while Figure 2a,b demonstrate percent changes in these endpoints over the study duration.

Radius:

In comparison to the HC group at baseline, AN-E+ had lower cortical area and thickness, cortical vBMD and tissue mineral density (TMD), trabecular vBMD, and failure load, and higher trabecular separation and cortical porosity. After adjusting for age and race, differences between groups persisted except for cortical vBMD, cortical TMD, trabecular separation, and cortical porosity. Following transdermal 17β-E2 replacement over 12 months, TMD, log cortical vBMD and log failure load increased in the AN-E+ group compared to HC (after controlling for baseline age and race). This increase in TMD, log cortical vBMD, and log failure load persisted even after adjusting for change in weight. Both groups showed within group increases in cortical area and thickness, trabecular area, cortical vBMD and TMD, and failure load over 12 months.

Tibia:

In comparison to the HC group at baseline, the AN-E+ group had lower cortical area and thickness, cortical vBMD, and trabecular number, and higher trabecular separation (after controlling for age and race). Following transdermal 17β-E2 replacement over 12 months, changes in cortical and trabecular parameters and bone strength estimates were similar in AN-E+ compared to HC. Both groups showed a within group increase in cortical vBMD and TMD. Of note, the within group increase in cortical area, cortical porosity and failure load, and decrease in trabecular area observed in HC over 12 months was not observed in AN-E+.

### 3.5. Adverse Events

Three participants experienced mild irritation at the site of patch application, which resolved after changing the site of application, two experienced irregular menses (increased frequency of menstruation) over a 1.5-month period, which resolved spontaneously, one participant experienced a mild increase in acne, one reported intermittent headache, one participant reported headache after taking two progesterone pills (which resolved the next morning), and three participants reported mild headaches following phlebotomy.

## 4. Discussion

This is the first study to demonstrate that transdermal physiologic estrogen replacement in adolescent girls and young women with anorexia nervosa results in similar changes in bone geometry, structure, and strength estimates at the distal radius and distal tibia as observed in healthy normal-weight normo-estrogenic girls. Further, physiologic estrogen replacement led to greater increases in aBMD at the lumbar spine, and in whole-body BMD than observed in HC suggesting some catch-up bone accrual. We have previously reported changes in aBMD after physiologic estrogen replacement in a younger and different cohort, however, that did not include assessments of changes in vBMD, bone structure or strength estimates [10].

Low BMD [2] and decreased bone accrual [4,5] are characteristic of AN during adolescence, when normal estrogen status is critical for achieving peak bone mass. The estrogen deficiency that often accompanies AN adversely affects both trabecular (lumbar spine) and cortical (total hip and whole-body) bone sites [17]. Consistent with this, adolescent and young adult women with anorexia nervosa in this cohort had lower aBMD at the lumbar spine, total hip, and whole body than HC. Physiologic transdermal estrogen replacement led to greater bone accrual at the lumbar spine and whole body compared to healthy normal-weight, normoestrogenic young women, suggesting that some catch-up growth can occur with estrogen replacement. This finding differs from what we have previously reported in a younger cohort, where physiologic estrogen replacement in girls with anorexia nervosa led to similar but not incremental bone accrual compared to healthy controls [10]. This may be consequent to the younger age of that AN cohort as compared to our current cohort, and/or differential changes in body composition over the duration of each study [10]. Because weight gain is closely associated with increases in BMD in adolescents with AN [5], we controlled for change in weight in this study, and noted that changes in aBMD at the lumbar spine and whole body remained significant, suggesting an independent effect of estrogen replacement beyond weight gain in the anorexia nervosa group.

Importantly, deficits in cortical and trabecular vBMD, structure, and strength at peripheral skeletal sites, as measured by HRpQCT, contribute to fracture risk independently of aBMD [18]. Girls with AN with estrogen deficiency have impaired bone area, geometry, structure, and strength estimates at the distal radius and tibia [7,8], and these changes have been reported to precede reductions in aBMD [9]. Consistent with this, our AN cohort had lower vBMD, suboptimal bone structure, and lower strength estimates than HC.

We show for the first time that physiologic estrogen replacement over 12 months improves cortical vBMD and TMD at the non-weight bearing radius and weight bearing tibia in adolescent girls and young adult women with anorexia nervosa to exceed or approximate changes observed in healthy, normal-weight, normoestrogenic controls over the same duration. Further, changes in radius geometry induced by physiologic estrogen replacement over 12 months approximate changes observed in controls. Neither group demonstrated significant changes in bone structure over time. The observed increment in TMD, cortical vBMD, and failure load at the distal radius (independent of change in weight) in AN girls compared to HC further underscores the importance of transdermal estrogen replacement in optimizing cortical bone mineralization and strength estimates at this site in AN.

Our study does have some limitations. Our inclusion and exclusion criteria may have resulted in the enrollment of a sample of adolescent girls and young women with AN that is not completely representative of the general population with this disorder. However, these criteria were necessary to ensure that the study design was rigorous and to minimize risk to study participants. Further, the duration of intervention was only a year and we did not examine the impact of physiologic estrogen replacement on fracture risk. Future studies with a larger sample size (including one that is more representative of the AN population) and a longer duration of follow-up are necessary to determine long-term trajectories of bone outcomes following transdermal estrogen replacement.

In conclusion, this is the first study evaluating the effect of transdermal physiologic estrogen replacement over 12 months on measures of vBMD, bone geometry, structure, and strength estimates in adolescents and young adult women with AN, with our data indicating that this strategy for estrogen replacement overall mimics or exceeds effects of endogenous estrogen on bone outcomes, thus improving skeletal health and potentially reducing fracture risk.

## Figures and Tables

**Table 1:** Baseline characteristics and change in clinical characteristics, anthropometric measures, and body composition over 12 months for AN-E+ -17β-E2 patch and HC groups.

Clinical Characteristics	Baseline Measures		*p* Value	Change over 12 Months		*p*-Value
	AN-E+ (*n* = 23)	HC (*n* = 27)		AN-E+ (*n* = 23)	HC (*n* = 27)	
Age (years)	19.29 ± 0.51	19.40 ± 0.47	0.876	–	–	–
Age at AN Diagnosis (years)	16.23 ± 0.48	–	–	–	–	–
Duration of Amenorrhea since Last Menses (months)	7.77 ± 2.72	–	–	–	–	–
Age of Menarche (years)	13.17 ± 0.35	12.51 ± 0.24	0.117	–	–	–
Race (White/African American/Others) (%)	21/0/2	45/3/10	**0.004**	–	–	–
Height (cm)	165.14 ± 1.28	164.40 ± 1.32	0.830	0.04 ± 0.14	0.37 ± 0.10 *	0.065
Weight (kg)	50.90 ± 1.37	59.35 ± 1.57	**0.0002**	1.78 ± 0.82 *	0.80 ± 0.42	0.305
BMI (kg/m^2^)	18.75 (17.53, 19.97)	21.27 (20.20, 24.43)	**<0.0001**	0.48 (−0.49, 1.83) *	0.16 (−0.41, 0.64)	0.066
BMI z-score	−1.13 (−1.58, −0.38)	−0.08 (−0.40, 0.84)	**<0.0001**	0.30 (−0.05, 0.80) *	−0.02 (−0.20, 0.05)	0.100
% Median BMI	86.83 (81.97, 94.58)	98.85 (94.04, 115.46)	**<0.0001**	2.26 (−2.19, 8.77) *	−0.22 (−2.36, 1.0)	**0.019**
Total Weight Bearing Activity (hours/week)	4.79 (1.64, 7.85)	1.13 (0, 3.18)	**0.001**	−0.48 (−3.99, 2.71)	0 (−0.56, 0.92)	0.630
**Body Composition (by DXA)**						
Total Lean Mass (kg)	37.69 ± 0.84	40.41 ± 1.06	0.0553	1.32 ± 0.44 *	−0.30 ± 0.28	**0.003**
% Lean Mass	72.90 ± 1.02	66.50 ± 0.91	**<0.0001**	−1.32 ± 1.11	−1.04 ± 0.48	0.803
Total Fat Mass (kg)	12.42 ± 0.76	18.40 ± 0.87	**<0.0001**	1.41 ± 0.83	0.84 ± 0.39	0.528
% Fat Mass	23.89 ± 1.03	33.31 ± 1.07	**<0.0001**	1.12 ± 1.27	−2.26 ± 0.80 *	**0.026**

Means ± SEM or median (interquartile range); Significant *p* values are bolded. BMI: body mass index. HC: healthy controls. * *p* < 0.05 for within group change over 12 months.

**Table 2:** Baseline levels and changes in biochemical parameters over 12 months.

Biochemical Parameters	Baseline Measures		*p*-Value	Change over 12 Months		*p*-Value
	AN-E+ (*n* = 23)	HC (*n* = 27)		AN-E+ (*n* = 23)	HC (*n* = 27)	
P1NP (ug/L)	100.99 ± 10.28	79.06 ± 7.13	0.068	−10.39 ± 9.47	−12.07 ± 4.45 *	0.983
NTX (nM BCE)	15.69 ± 5.56	16.62 ± 8.82	0.672	−3.40 ±1.43 *	−2.43 ± 1.24	0.610
Calcium (mg/dL)	9.44 ± 0.06	9.07 ± 0.09	**0.002**	−0.16 ± 0.08 *	0.39 ± 0.08 *	**<0.0001**
25OHD (ng/mL)	41.04 ± 3.75	25.93 ± 2.44	**0.0002**	−9.27 ± 3.42 *	3.53 ± 2.41	**0.002**
PTH (pg/mL)	21.69 ± 2.13	31.30 ± 2.53	**0.006**	0.16 ± 2.59	−1.44 ± 3.29	0.725
IGF-1 (ng/mL)	260.0 ± 17.5	286.0 ± 18.8	0.318	−30.2 ± 14.7	−30.1 ± 14.8	0.994
IGF-1 Z-score	−0.42 ± 0.24	−0.05 ± 0.12	0.193	−0.08 ± 0.17	−0.09 ± 0.16	0.973

Means ± SEM or Median (interquartile range); Significant *p* values are bolded. 25OHD: 25-hydroxycholecalciferol; PTH: Parathyroid hormone; P1NP: N-terminal propeptide of type 1 procollagen; NTX: N-terminal cross-linking telopeptide; IGF-1: Insulin-like growth factor. * *p* < 0.05 for within group changes.

**Table 3:** Baseline and 12-month change in areal BMD assessed by DXA.

Areal Bone Mineral Density (by DXA)	Baseline Measures		*p*-Value	Change over 12 Months		*p*-Value Adjusted for Baseline Age and Race	*p*-Value Adjusted for Baseline Age, Race and Change in Weight
	AN-E+ (*n* = 23)	HC (*n* = 27)		AN-E+ (*n* = 23)	HC (*n* = 27)		
Femoral Neck BMD (g/cm^2^)	0.74 ± 0.02	0.84 ± 0.02	**0.0016 ^†^**	0.01 ± 0.008	0.003 ± 0.004	0.501	0.417
Femoral Neck Z-score	−1.40 ± 0.23	−0.37 ± 0.18	**0.0006 ^†^**	0.37 ± 0.09 *	0.09 ± 0.05	**0.009**	**0.017**
Total Hip BMD (g/cm^2^)	0.85 ± 0.03	0.96 ± 0.02	**0.0005 ^†^**	0.020 ± 0.007 *	0.006 ± 0.004	0.072	0.084
Total Hip Z-score	−0.82 ± 0.22	0.004 ± 0.16	**0.0030 ^†^**	0.15 ± 0.07 *	0.04 ± 0.03	0.081	0.119
Lumbar BMD (g/cm^2^)	0.84 ± 0.03	0.97 ± 0.02	**0.0006 ^†^**	0.042 ± 0.008 *	0.008 ± 0.005	**0.001**	**0.004**
Lumbar BMD Z-Score	−1.60 ± 0.24	−0.53 ± 0.20	**0.0013 ^†^**	0.28 ± 0.08 *	0.04 ± 0.06	**0.023**	0.054
Whole Body BMD (g/cm^2^)	0.97 ± 0.01	1.05 ± 0.02	**0.0002 ^†^**	0.020 ± 0.001 *	−0.002 ± 0.004	**0.011**	**0.0003**
Whole Body BMD Z-score	−1.57 ± 0.19	−0.64 ± 0.17	**0.0006 ^†^**	0.12 ± 0.07	−0.11 ± 0.06	0.057	**0.004**

Means± SEM or Median (interquartile range); Significant *p* values are bolded. BMD: bone mineral density. ^†^ *p* < 0.05 after adjusting for age and race. * *p* < 0.05 for within group changes.

**Table 4:** Baseline and 12-month change in bone parameters assessed by HRpQCT.

HRpQCT Variables	Baseline Measures		*p* Value	Change over 12 Months		*p* Value Adjusted for Baseline Age and Race	*p* Value Adjusted for Baseline Age, Race and Change in Weight
Radius	AN-E+ -17β-E2 (*n* = 21)	HC (*n* = 26)		AN-E+ -17β-E2 (*n* = 21)	HC (*n* = 26)		
Cortical Area (mm^2^)	44.10 (35.50, 50.9)	56.45 (47.23, 62.90)	**0.012 ^†^**	2.25 (−0.25, 7.00) *	1.35 (−0.18, 2.33) *	0.719	0.891
Trabecular area (mm^2^)	222.6 (199.5, 239.5)	206.8 (171.8, 247.7)	0.634	−2.00 (−3.70, −0.05) *	−1.30 (−2.63, 0.60) *	0.647	0.715
Cortical Thickness (mm)	0.68 (0.54, 0.84)	0.84 (0.71, 0.94)	**0.012 ^†^**	0.05 (0.00, 0.11) *	0.02 (−0.003, 0.04) *	0.595	0.746
Cortical vBMD (mgHA/cm^3^)	816.3 (770.9, 842.6)	862.5 (820.3, 881.9)	**0.005**	16.80 (6.45, 37.85) *	10.40 (3.88, 16.53) *	**0.018** **^a^**	**0.029** **^a^**
Cortical TMD (mgHA/cm^3^)	918.6 (884.6, 949.1)	948.1 (907.4, 974.2)	**0.048**	19.8 (10.7, 30.4) *	10.2 (6.0, 14.0) *	**0.015**	**0.010**
Trabecular vBMD (mgHA/cm^3^)	153.7 (124.1, 176.8)	174.1 (145.1, 199.8)	**0.047 ^†^**	3.20 (−4.45, 4.85)	−0.35 (−1.78, 2.63)	0.904	0.554
Number of Trabeculae (1/mm)	1.90 ± 0.04	2.02 ± 0.05	0.065	−0.04 ± 0.04	−0.02 ± 0.03	0.312	0.399
Trabecular Thickness (mm)	0.06 (0.06, 0.08)	0.07 (0.06, 0.08)	0.130	0.003 (−0.003, 0.006)	0.002 (−0.002, 0.005)	0.564	0.360
Trabecular Separation (mm)	0.46 ± 0.01	0.43 ± 0.01	**0.042**	0.009 ± 0.011	0.004 ± 0.008	0.302	0.377
Cortical Porosity (%)	1.0 (0.7, 1.3)	0.7 (0.4, 1.3)	**0.040**	−0.1 (−0.3, 0.1)	0.1 (−0.1, 0.5)	0.122	0.198
Failure Load (kN)	3.32 (2.56, 4.18)	4.05 (3.66, 4.59)	**0.011 ^†^**	0.14 (−0.05, 0.32) *	0.04 (−0.03, 0.14) *	**0.004** **^a^**	**0.008** **^a^**
**Tibia**	**AN-E+ -17β-E2** **(*n* = 23)**	**HC** **(*n* = 27)**		**AN-E+ -17β-E2** **(*n* = 23)**	**HC** **(*n* = 27)**		
Cortical Area (mm^2^)	102.7 (88.8, 120.1)	125.0 (105.9, 139.1)	**0.001 ^†^**	0.7 (−0.6, 3.3)	0.5 (0.0, 1.4) *	0.769	0.774
Trabecular Area (mm^2^)	554.1 (474.7, 585.9)	545.3 (457.4, 583.5)	0.718	0.1 (−1.3, 1.0)	−0.5 (−1.2, 0.3) *	0.728	0.796
Cortical Thickness (mm)	1.06 ± 0.03	1.24 ± 0.04	**0.004 ^†^**	0.01 ± 0.04	0.00 ± 0.00	0.968	0.980
Cortical vBMD (mgHA/cm^3^)	859.8 ± 7.2	884.4 ± 7.4	**0.023 ^†^**	9.6 ± 1.9 *	9.2 ± 2.7 *	0.900	0.985
Cortical TMD (mgHA/cm^3^)	954.4 (916.4, 983.1)	981.9 (959.1, 1006.2)	**0.009**	8.1 (3.5, 22.5) *	4.8 (−0.4, 11.2) *	0.120	0.917
Trabecular vBMD (mgHA/cm^3^)	174.9 (153.9, 194.7)	184.0 (167.8, 216.4)	0.119	1.0 (−2.1, 2.1)	0.2 (−2.0, 2.2)	0.941	0.940
Number of Trabeculae (1/mm)	1.73 (1.62, 1.84)	1.92 (1.81, 2.12)	**0.002 ^†^**	0.03 (−0.06, 0.08)	−0.04 (−0.16, 0.04)	0.514	0.975
Trabecular Thickness (mm)	0.08 (0.07, 0.09)	0.08 (0.07, 0.10)	0.601	−0.002 (−0.006, 0.003)	0.001 (−0.003, 0.004)	0.415	0.267
Trabecular Separation (mm)	0.49 (0.46, 0.54)	0.43 (0.40, 0.48)	**0.003 ^†^**	−0.01 (−0.02, 0.02)	0.01 (−0.01, 0.04)	0.485	0.966
Cortical Porosity (%)	2.5 (1.9, 3.4)	1.4 (0.9, 2.6)	**0.010**	−0.1 (−0.3, 0.2)	0.3 (−0.1, 1.3) *	0.194	0.306
Failure Load (kN)	9.68 (8.74, 11.07)	10.79 (9.80, 12.04)	0.091	−0.03 (−0.23, 0.18)	0.12 (−0.01, 0.25) *	0.177	0.368

Means ± SEM or Median (interquartile range); Significant *p* values are bolded. vBMD: volumetric bone density; TMD-tissue mineral density. † *p* < 0.05 after adjusting for age and race. ^a^ *p* < 0.05 on log conversion for right skewed data. * p < 0.05 for within group changes.

## Data Availability

The datasets used and analyzed during the current study are available from the principal investigators of the study on reasonable request.

## Abbreviations

AN	Anorexia Nervosa
DSM	Diagnostic and Statistical manual of Mental Disorders
BMD	Bone Mineral Density
vBMD	Volumetric Bone Mineral Density
aBMD	Areal Bone Mineral Density
HC	Healthy Control
17βE2	17-beta Estradiol
BMI	Body Mass Index
% mBMI	Percentage Median Body Mass Index
% mBW	Percentage Median Body Weight
% mBW-Ht	Percentage Median Body Weight for Height
FSH	Follicle Stimulating Hormone
TSH	Thyroid Stimulating Hormone
DXA	Dual X-ray Absorptiometry
HRpQCT	High Resolution Peripheral Quantitative Computed Tomography
TMD	Tissue Mineral Density
ECA	Extended Cortical Analysis
µFEA	Micro Finite Element Analysis
SEM	Standard Error Mean
25OHD	25-hydroxy Vitamin D
P1NP	N-terminal Propeptide of Type 1 Procollagen
NTX	N-terminal Cross-linking Telopeptide
PTH	Parathyroid Hormone
IGF-1	Insulin-like Growth Factor-1
